# Drug effects in multiple rounds of cell division monitored by flow cytometry

**DOI:** 10.1186/1476-9255-12-S1-O6

**Published:** 2015-04-16

**Authors:** Derek Davies

**Affiliations:** 1 FACS Laboratory, London Research Institute, Cancer Research UK, 44 Lincolns Inn Fields, London, WC2A 3LY, UK

## 

Flow cytometry, and the more recently introduced imaging flow cytometry, are ideal tools for following the cell cycle in mammalian cells. One of the first applications of flow cytometry was looking at DNA content via single parameter fluorescence of a DNA-binding dye such as propidium iodide or DAPI but this only provides a snapshot of cells at one point in time. More sophisticated techniques are needed to investigate multiple rounds of division and these include the addition of a nucleotide analog such as bromodeoyurdine (BrdU) which can follow cells through 1-2 rounds of cell division; the BrdU-Hoechst quenching technique which can follow 3-4 rounds of cell division or the use of a dye dilution technique using protein binding dyes such as CFSE or the CellTrace dyes or lipid-binding dyes such as the PKH dyes. We recently developed a method to study cell cycle analysis in multiple rounds of division using a combination of CellTrace Violet for round of division, propidium iodide for DNA content and staining for phoshpoH3 to identify mitotic cells [1]. This enables us to follow cells through up to 6 rounds of cell division. By adding drugs that interfere with the cell cycle either by inhibiting DNA synthesis (etoposide) or that inhibit mitosis (Nocodazol) we are able to look at the effect in cells at a known point in division history without the need for blocking the cells beforehand. This gives a true sense of how individual cells relate to one another. In addition, by using imaging flow cytometry we are able to sub-divide the individual phases of mitosis which can give further information on the action of cell-cycle inhibiting drugs and may allow this method to be used to give mechanistic information of the action of candidate anti-cancer drugs.
